# Ultra-confined Propagating Exciton–Plasmon Polaritons Enabled by Cavity-Free Strong Coupling: Beating Plasmonic Trade-Offs

**DOI:** 10.1186/s11671-022-03748-7

**Published:** 2022-11-18

**Authors:** Yipei Wang, Aoning Luo, Chunyan Zhu, Zhiyong Li, Xiaoqin Wu

**Affiliations:** 1 Key Laboratory of Optoelectronic Technology and Systems (Ministry of Education), College of Optoelectronic Engineering, Chongqing University, Chongqing, 400044 People’s Republic of China; 2 State Key Laboratory of Modern Optical Instrumentation, College of Optical Science and Engineering, Zhejiang University, Hangzhou, 310027 People’s Republic of China; 3 Jiaxing Key Laboratory of Photonic Sensing and Intelligent Imaging, Jiaxing, 314000 People’s Republic of China; 4 Jiaxing Intelligent Optics and Photonics Research Center, Jiaxing Research Institute Zhejiang University, Jiaxing, 314000 People’s Republic of China

**Keywords:** Strong coupling, Exciton–plasmon polaritons, Waveguiding, Transition metal dichalcogenides, Metal nanowires

## Abstract

Hybrid coupling systems consisting of transition metal dichalcogenides (TMD) and plasmonic nanostructures have emerged as a promising platform to explore exciton–plasmon polaritons. However, the requisite cavity/resonator for strong coupling introduces extra complexities and challenges for waveguiding applications. Alternatively, plasmonic nano-waveguides can also be utilized to provide a non-resonant approach for strong coupling, while their utility is limited by the plasmonic confinement-loss and confinement-momentum trade-offs. Here, based on a cavity-free approach, we overcome these constraints by theoretically strong coupling of a monolayer TMD to a single metal nanowire, generating ultra-confined propagating exciton–plasmon polaritons (PEPPs) that beat the plasmonic trade-offs. By leveraging strong-coupling-induced reformations in energy distribution and combining favorable properties of surface plasmon polaritons (SPPs) and excitons, the generated PEPPs feature ultra-deep subwavelength confinement (down to 1-nm level with mode areas ~ 10^–4^ of *λ*^2^), long propagation length (up to ~ 60 µm), tunable dispersion with versatile mode characters (SPP- and exciton-like mode characters), and small momentum mismatch to free-space photons. With the capability to overcome the trade-offs of SPPs and the compatibility for waveguiding applications, our theoretical results suggest an attractive guided-wave platform to manipulate exciton–plasmon interactions at the ultra-deep subwavelength scale, opening new horizons for waveguiding nano-polaritonic components and devices.

**Supplementary Information:**

The online version contains supplementary material available at 10.1186/s11671-022-03748-7.

## Introduction

As an intriguing regime of the light–matter interaction, strong coupling between excitons and photons with the formation of polaritons enables great possibilities to modify the properties of the coupled systems, offering numerous opportunities for both fundamental research and technological applications including Bose–Einstein condensation [1], low-threshold lasing [2], ultrafast modulation and switching [3, 4], and all-optical logic operation [5]. Recently, owing to their remarkable excitonic properties such as large binding energies and strong oscillator strengths [6], monolayer transition metal dichalcogenides (TMDs) are emerging as promising candidate two-dimensional (2D) materials to sustain the exciton resonance for reaching the strong coupling regime. By combining them to plasmonic nanostructures with ultra-tight optical confinement, the great size mismatch between the optical field and the ultra-thin thickness of monolayer TMDs can be bridged, providing the unprecedented ability to explore the strong plasmon–exciton interaction at the deep subwavelength scale [7].

Generally, in the plasmonic-TMD system, the key for achieving strong coupling is to ensure a sufficiently large coupling strength that overcomes the overall damping of the coupled system. And a common strategy is to utilize tightly confined cavity modes or localized surface plasmon resonances (LSPRs), which have been previously realized by introducing plasmonic cavities or resonators including metallic F-P cavities [8], periodic structures [9–11], plasmonic dimers [12, 13], single nanoparticles [14–18], and nanogap resonators formed by nanoparticle-over-mirror configurations [19–21]. However, the requisite cavity/resonator may introduce extra complexities [22, 23] and challenges for flexible mode engineering [24], on-chip integration [25], and remote exciton–polariton transportations [26] for waveguiding applications.

On the other hand, besides the cavity modes and LSPRs, propagating modes can also be utilized to provide a non-resonant approach for strong coupling [23, 27–30], but have received little attention in the plasmonic nano-waveguiding system. As one of the simplest one-dimensional (1D) nano-waveguides to support propagating surface plasmon polaritons (SPPs), metal nanowires (MNWs) possess unique advantages including excellent compatibilities to on-chip nanophotonics [31] and deep subwavelength confinement (e.g., ~ 10^–2^ ~ 10^–3^ of *λ*^2^) [32–35] for promoting light–matter interactions, offering a potential guided-wave platform for strong coupling. However, the utility of MNWs is limited by the well-known trade-off between the energy confinement and the loss of SPPs [33, 35]. In addition to the confinement-loss trade-off, another fundamental hurdle is the trade-off between confinement and momentum mismatch to photons [36], leading to challenges for efficient photon-SPP conversions and consequently weakened compatibilities for integrated hybrid components and devices.

Here, based on a MNW-TMD system, we theoretically propose a cavity-free strong coupling approach for generating ultra-confined propagating exciton–plasmon polaritons (PEPPs) that beat the plasmonic confinement-loss and confinement-momentum trade-offs. We show that the strong coupling between SPPs in a single MNW and excitons in a monolayer WS_2_ results in a back-bending dispersion with the complex momentum and an anti-crossing dispersion with the complex frequency, exhibiting large Rabi splitting energies with tunability. Due to the strong-coupling-induced reformation in the energy distribution, the generated PEPPs are much more confined than the original SPPs in MNWs, offering the possibility to realize a full width at half maximum of the energy distribution at the ultra-deep subwavelength scale (~ 1 nm). Meanwhile, as a mixture of SPPs and excitons, PEPPs are highly versatile that can be manipulated to exhibit exciton-like character with extremely tight confinement (~ 10^–4^ of *λ*^2^) or SPP-like character with high quality and long propagation distance (up to ~ 60 µm). More importantly, we also show that PEPPs represent another class of waveguiding polaritons with much more efficient confinement-loss and confinement-momentum trade-offs that outperforms the original SPPs, which may offer new opportunities for waveguiding polaritonic applications such as ultra-compact integrated circuits and high-performance polaritonic devices.

## Methods

**Fig. 1:**
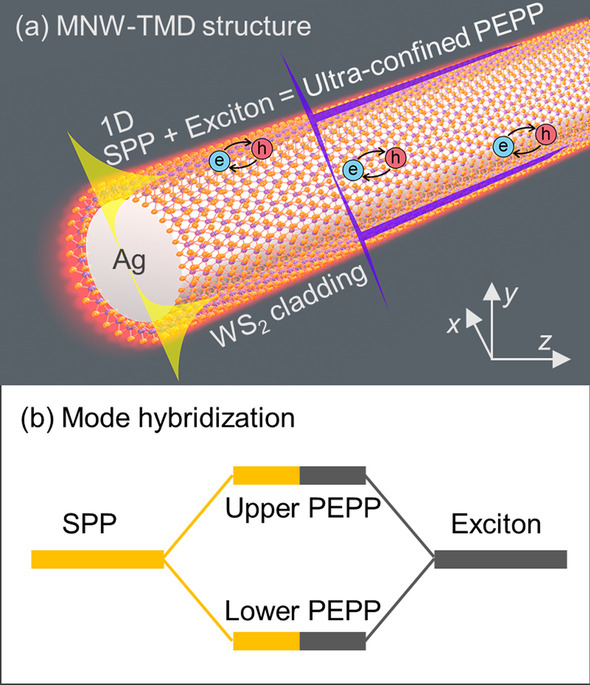
**a** Schematic illustration of the MNW-TMD structure consisting of a Ag MNW and a monolayer WS_2_ cladding. The MNW cross section is at the *x*–*y* plane, while the propagation direction is along the *z* axis. **b** Schematic illustration of the hybridization of the SPPs and excitons

The proposed MNW-TMD structure consists of a single MNW waveguide with a monolayer TMD cladding, which is schematically plotted in Fig. 1a. The MNW is assumed to have a uniform diameter with a smooth surface. In such configuration, the tightly confined SPP with strong field enhancement around the MNW-TMD interface facilitates the plasmon–exciton interaction. As a model system for theoretical investigation, a WS_2_-clad Ag MNW is selected, in which the permittivities of WS_2_ (*ε*_WS2_) and Ag (*ε*_Ag_) are described by a Lorentz oscillator model [25] and an effective Drude model [37], respectively (see supporting information for details). The thickness of the WS_2_ layer is assumed to be 1 nm [12]. For simplicity and facilitating strong coupling, we only focus on the coupling of excitons to the fundamental mode in the Ag MNW, since the fundamental mode is more confined than the other order ones [35, 38], and the single-mode operation is favorable and can be readily realized in many applications [32, 33, 35, 39].

For theoretical investigation of the strong coupling and the formed PEPP in the proposed coupling system, the wave equations are numerically solved in both complex-frequency (complex-*ω*) and complex-momentum (complex-*k*) planes to provide a comprehensive analysis. As to the complex-*ω* solution, the real (Re(*ω*)) and imaginary (Im(*ω*)) parts represent the eigenfrequency and the temporal damping of the PEPP in the coupled system. And for the complex-*k* solution, the real (Re(*k*)) and imaginary (Im(*k*)) parts correspond to the propagation constant and the spatial damping along the propagation direction. Besides solutions from the wave equation, the PEPP can also be decomposed into the exciton resonance in the WS_2_ and the uncoupled SPP mode from the hybridization point of view (Fig. 1b). The bare SPP mode without coupling to excitons (refer to the SPP in further text) is obtained by using the non-resonant background permittivity *ε*_*b*_ of the WS_2_ with its oscillator strength being zero (see numerical methods in supporting information for details).

## Results and Discussion

### Cavity-Free Strong Coupling Between Excitons and 1D-SPPs

**Fig. 2:**
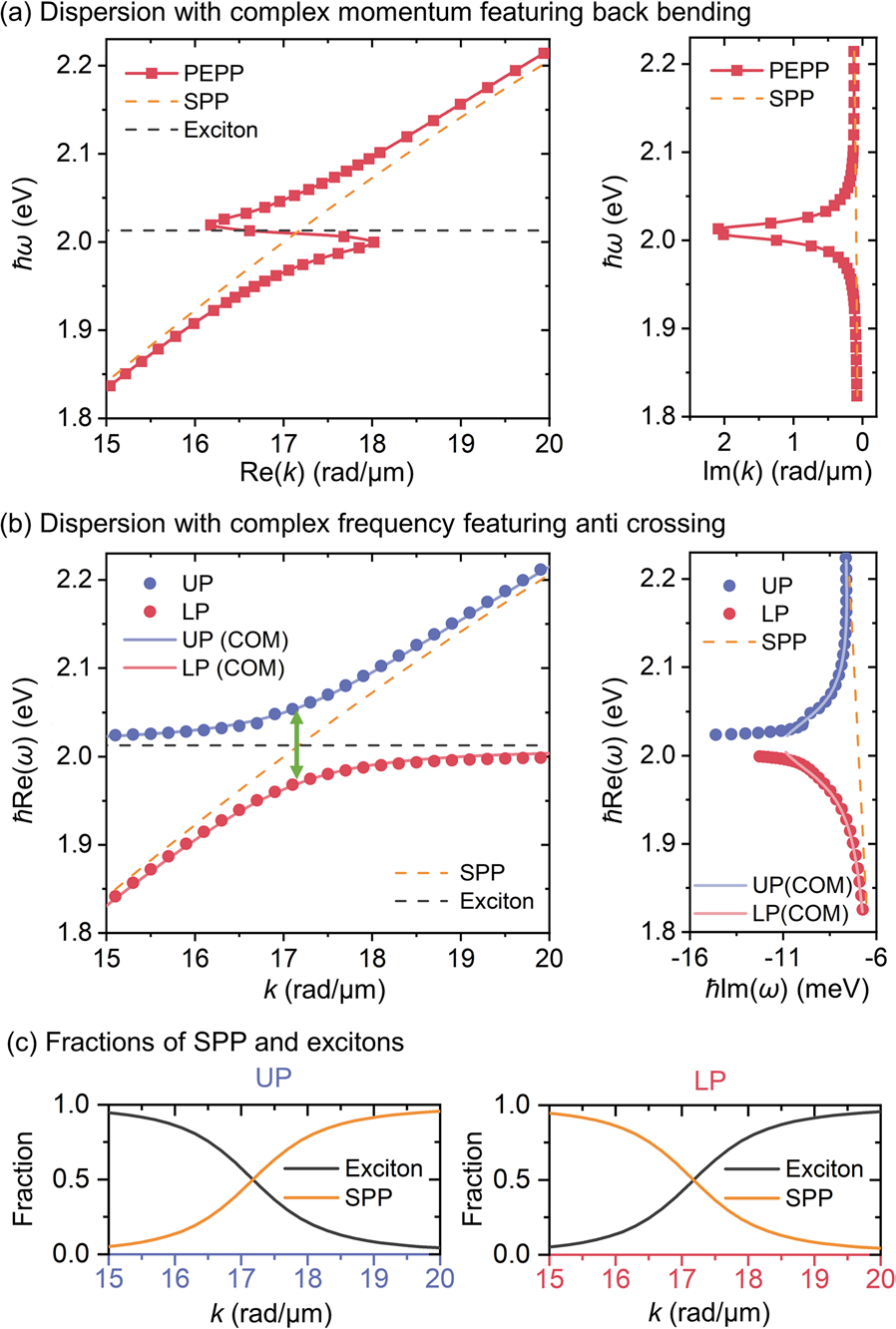
Strong coupling between excitons and SPPs in a WS_2_-clad Ag MNW. **a** Solutions in the complex-momentum (complex-*k*) plane by feeding real-valued *ω*. Left: dispersion relation in terms of *ħω* vs. Re(*k*) featuring back-bending. Right: trajectories of *ħω* and Im(*k*). Red squared, yellow dashed, and black dashed lines: PEPP, SPP, and excitons. **b** Solutions in the complex frequency (complex-*ω*) plane by feeding real-valued *k*. Left: dispersion relation in terms of *ħ*Re(*ω*) versus *k* featuring anti-crossing. Right: trajectories of *ħ*Re(*ω*) and *ħ*Im(*ω*). The numerically obtained result for the PEPP is denoted by blue (upper branch, UP) and red dots (lower branch, LP), showing excellent agreement to the result obtained from the coupled-oscillator model (COM, blue and red lines) with a Rabi splitting energy ~ 85.7 meV (green arrow). Yellow and black dashed lines: SPP and excitons. **c** Fractions of excitons (black lines) and the SPP (yellow lines) in the PEPP. Left: upper branch. Right: lower branch. The diameter for Ag MNW here is 50 nm

Figure 2A gives the complex-*k* solution of the PEPP with the MNW diameter of 50 nm. As to its dispersion curve (*ħω* vs. Re(*k*), left panel), the hybridization of the exciton (black dashed line) and the SPP mode (orange dashed line) gives rise to the anomalous dispersion in the vicinity of the exciton resonance with a significant back-bending feature, clearly indicating strong coupling [23, 27, 29]. Meanwhile, when *ω* is approaching the exciton resonance, Im(*k*) of the PEPP dramatically increases (Fig. 2a, right panel), resulting in a drastic reduction in its propagation length that will be discussed later in waveguiding properties. For comprehensive characterization, Fig. 2b presents the corresponding complex *ω* solutions of the PEPP. Instead of the continuous dispersion curve in the complex-momentum plane, the dispersion in terms of *ħ*Re(*ω*) versus *k* (Fig. 2b, left panel) exhibits two asymptotic branches (upper branch: blue dots; lower branch: red dots) disconnected by a polaritonic gap around the exciton resonance, manifesting itself in an anti-crossing behavior with the Rabi splitting energy (*ħ*Ω_*R*_) of ~ 85.7 meV at the zero-detuning (green double arrow). Compared to the SPP, the PEPP exhibits a “left-pulling” trend in the complex-*ω* trajectory (Fig. 2b, right panel) and becomes highly damped around the excitonic resonance which corresponds well to other propagating polaritons previously reported [23]. Note that the aforementioned back-bending and anti-crossing behaviors in dispersions are not inconsistent with each other [23, 29, 40], and they are actually the results obtained at a given frequency or momentum and can be both experimentally measured by momentum- and frequency-resolved spectroscopy [40].

For further verification, we approximate our system to the coupled-oscillator model (COM) [41]:

1 $$\left( {\begin{array}{*{20}{c}} {{\omega_{{\text{SPP}}}} - i\frac{{{\gamma_{{\text{SPP}}}}}}{2}}&{\frac{{\Omega_R}}{2}} \\ {\frac{{\Omega_R}}{2}}&{{\omega_{{\text{ex}}}} - i\frac{{{\gamma_{{\text{ex}}}}}}{2}} \end{array}} \right){\left( \begin{gathered} \alpha \hfill \\ \beta \hfill \\ \end{gathered} \right)_\pm } = {\omega_\pm }{\left( \begin{gathered} \alpha \hfill \\ \beta \hfill \\ \end{gathered} \right)_\pm },$$

from which the eigenvalues *ω*_±_ of the PEPP can be analytically obtained through diagonalization of the Hamiltonian matrix, and the eigenvectors $(\alpha ,\beta )_\pm^T$ are determined for revealing contributions from SPPs (|*α*_±_|^2^) and excitons (|*β*_±_|^2^). Here, *ω*_SPP_ and *γ*_SPP_ are eigenfrequency and damping frequency of the SPP mode (i.e., *ω*_SPP_ = Re(*ω*), *γ*_SPP_ = 2|Im(*ω*)| extracted from the orange dashed line in Fig. 2b). *ω*_ex_ and *γ*_ex_ are exciton resonance frequency and damping frequency of the WS_2_ material. With the above parameters, the analytically obtained results using the COM (solid line in Fig. 2b) exhibit excellent agreements to numerical simulations for both of the dispersion curve and the complex-*ω* trajectory. Note that Im(*ω*) around the excitonic resonance from the simulation is slightly larger than the one analytically obtained, which may be attributed to the extra dissipation caused by the extremely tight confinement [42, 43] near the excitonic resonance that is not considered in the analytical model. To claim the strong coupling, the strict criterion *ħ*Ω_*R*_ > *ħ*(*γ*_SPP_ + *γ*_ex_)/2 [41] is well fulfilled comparing the Rabi energy *ħ*Ω_*R*_ of ~ 85.7 meV to the overall damping in the system *ħ*(*γ*_SPP_ + *γ*_ex_)/2 of ~ 18 meV. As to fractions of SPPs and excitons in PEPPs, they are equally contributed (|*α*_±_|^2^ =|*β*_±_|^2^ = 0.5) for both upper and lower branches at the zero-detuning (Fig. 2c) corresponding to *ħω*_±_ = 2.057 (~ 603 nm in wavelength *λ*) and 1.971 eV (*λ* ~ 629 nm), respectively. Within this range (1.971 eV < *ħω* < 2.057 eV), PEPPs are dominant by excitons in terms of |*β*_±_|^2^ > 0.5 (exciton-like) and they are otherwise SPP-like outside the range.

### Mode Characteristics and Waveguiding Properties of PEPPs

**Fig. 3:**
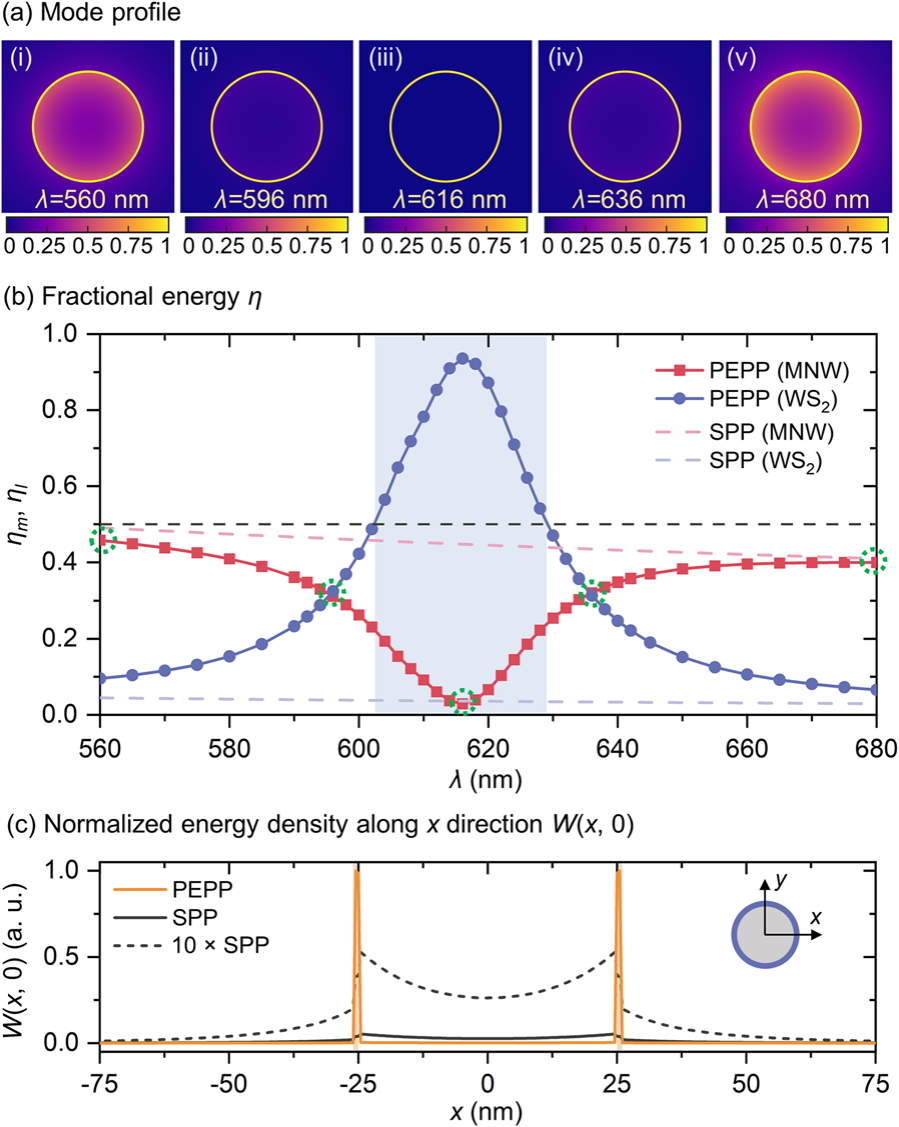
Mode characteristics and evolution with wavelengths (*λ*) of the PEPP. **a** Mode profiles in terms of energy density distributions, showing the mode transition between SPP-like and exciton-like characters, (**i**–**v**) *λ* = 560, 596, 616, 636, and 680 nm, respectively. **b** Quantitative characterization in terms of fractional energy inside the MNW (*η*_*m*_) and the WS_2_ layer (*η*_*l*_). *η*_*m*_ and *η*_*l*_ for PEPP (SPP) are denoted by squared red and dot blue lines (red and blue dashed lines), respectively. For the SPP, *η*_*l*_ is calculated by setting oscillator strength of the WS_2_ cladding to be zero. For the PEPP, black dashed line indicates *η*_*l*_ = 0.5, and blue-filling area indicates the exciton-like region. Green circles: *η*_*m*_ for PEPPs at the wavelengths of 560, 596, 616, 636, and 680 nm shown in **a**. Their corresponding *η*_*m*_ are ~ 0.46 **a**(**i**), ~ 0.32 **a**(**ii**), ~ 0.03 **a**(**iii**), ~ 0.32 **a**(**iv**), and ~ 0.40 **a**(**v**), respectively. **c** Normalized energy density along *x* direction *W*(*x*, 0) at *λ* = 616 nm. Yellow line: PEPP. Gray solid and dashed lines: SPP and its 10 times multiplication for clear visualization. Inset: coordinates on the cross section. The diameter for the Ag MNW here is 50 nm

The above results evidently show that SPPs in MNWs can be strongly coupled to excitons in the WS_2_ monolayer, creating PEPPs that are hybrid mixtures of SPPs and excitons. Due to the hybrid nature, the fractions of SPPs and excitons in PEPPs can be manipulated by the wavelength *λ* (which is discussed in Fig. 2c), offering opportunities to alter and even reform the energy distribution of PEPPs. To gain a deeper insight, Fig. 3a, b gives cross-sectional mode profiles and *λ*-dependent fractional energy distributions of PEPPs, in which the fractional energy inside the MNW (*η*_*m*_) and the WS_2_ layer (*η*_*l*_) is calculated via their corresponding energy density *W*(*x*, *y*) [44] integrations (using Additional file 1: Eq. S1). For reference, the corresponding fractional energy for the SPP is also provided (pale red and blue dashed lines, Fig. 3b). It can be seen that at wavelengths distant away from the excitonic resonance (e.g., *λ* = 560 and 680 nm, Fig. 3a(i), (v)), where the plasmon–exciton interaction is relatively weak, the PEPP shows a SPP-like mode character with a much larger *η*_*m*_ than *η*_*l*_ (e.g., *η*_*m*_ ~ 0.46 vs. *η*_*l*_ ~ 0.10 at *λ* = 560 nm). As wavelengths approach the excitonic resonance, *η*_*l*_ rapidly increases with more energy being pulled out from the MNW and mode profiles shifted to the WS_2_ layer (e.g., Fig. 3a(ii), (iv) for *λ* = 596 and 636 nm, *η*_*m*_ = *η*_*l*_ ~ 0.32). And at wavelengths of 603 and 629 nm, *η*_*l*_ increases to 0.5 (which also coincides very well with the calculated result from |*β*_±_|^2^ = 0.5), indicating that the PEPP mode enters the exciton-like region (blue-filling area, Fig. 3b). Finally, around the excitonic resonance wavelength (*λ* ~ 616 nm), *η*_*l*_ reaches its maximum (*η*_*l*_ ~ 0.94), enabling an extremely tight confinement with most of the energy inside the WS_2_ layer (Fig. 3a(iii)). To better visualize such strong-coupling-induced reformation in the energy distribution, Fig. 3c gives the normalized energy density along the *x* direction *W*(*x*, 0). Compared to the SPP, the PEPP further squeezes the energy into the atomic-thin 2D materials with the full width at half maximum (FWHM) of the energy distribution at the ultra-deep subwavelength scale (~ 1 nm), offering a promising route to enhance the light–matter interaction that may have great potentials for nonlinear applications.

**Fig. 4:**
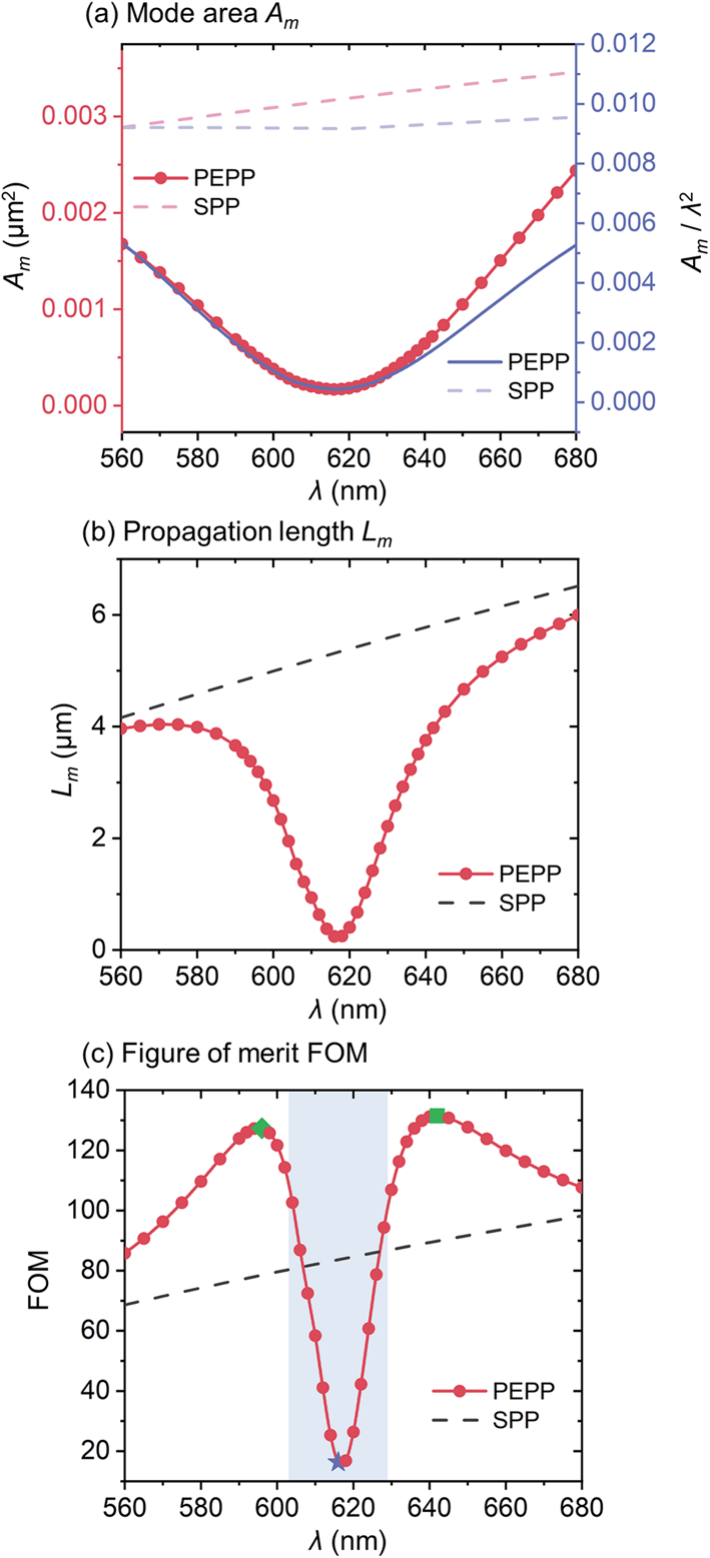
Waveguiding properties including **a** mode area *A*_*m*_ (left *y*-axis) and normalized mode area *A*_*m*_/*λ*^2^ (right *y*-axis), **b** propagation length *L*_*m*_, and **c** figure of merit (FOM) of the PEPP and SPP with a MNW diameter of 50 nm. Compared to the monotonic behaviors of the SPP, the PEPP is able to exert its full strength for energy confinement in the exciton-like region (blue-filling area in **c**), while is capable of achieving excellent FOM in the SPP-like region (non-filling area in **c**). Blue star: maximum confinement. Green diamond and square: local maximum FOMs. The diameter for the Ag MNW here is 50 nm

For further quantitative characterization of the confinement, Fig. 4a gives mode areas *A*_*m*_ (calculated using Additional file 1: Eq. S2) of the PEPP (red dotted line). As is shown, benefitted from the strong-coupling-induced reformation in the energy distribution, *A*_*m*_ of the PEPP is always much smaller than the SPP (red-dashed line), making it possible to realize an extremely small value down to 0.000169 µm^2^ (~ 4 × 10^–4^ of *λ*^2^, see right *y*-axis for the normalized mode area) that is only ~ 1/20 the size of the corresponding SPP. On the other hand, the propagation lengths *L*_*m*_ (calculated using Additional file 1: Eq. S3) are shown in Fig. 4b. The profound dip in the *L*_*m*_ curve with a drastic reduction from ~ 6 to 0.24 µm is due to the most energy distributed in the WS_2_ layer with higher absorption, which may have potential applications in all-optical switching and modulation. For other applications where the long-range propagation is desired, *L*_*m*_ of the PEPP can be manipulated by increasing the MNW diameter, while the strong coupling still holds valid (e.g., *L*_*m*_ =  ~ 60 µm can be achieved for a 400-nm-diameter MNW which will be discussed in the next section). Moreover, as a mixture, the PEPP inherits both properties of the SPP and the exciton, offering opportunities to achieve higher versatility and superior quality than the bare SPP. For demonstration, the calculated figure of merit (FOM = ${{L_m} \mathord{\left/ {\vphantom {{L_m} {(2\sqrt {{A_m}/\pi } }}} \right. \kern-\nulldelimiterspace} {(2\sqrt {{A_m}/\pi } }})$ [45]) of both PEPP and SPP is shown in Fig. 4c. Instead of the monotonic behaviors of the SPP, the FOM curve of the PEPP is divided into two types of regions (indicated by the blue-filling and non-filling areas) according to the mode characters (exciton-like and SPP-like). In the blue-filling zone where the exciton dominates, the PEPP is able to exert its full potential for confining energy at the ultra-deep subwavelength scale (e.g., at the maximum confinement wavelength of ~ 616 nm, blue star), while in the SPP-like region, the PEPP can offer higher FOM than SPP with two local maximum values of ~ 127 and 131 (at the maximum FOM wavelengths: ~ 596 and 642 nm, green diamond and square) around transitions of mode characters.

### Tunability in Rabi Splitting Energy with Tailored Dispersions

**Fig. 5:**
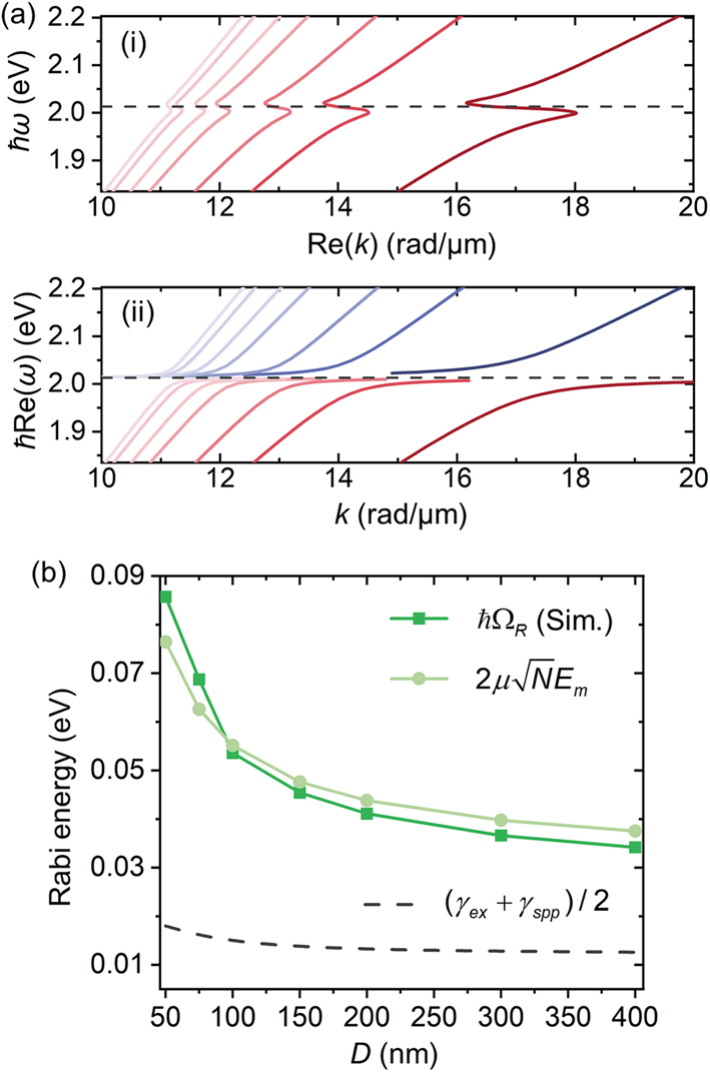
Tunability in Rabi splitting energy (*ħ*Ω_*R*_) with tailored dispersions. **a** Dispersion relations in terms of (**i**) *ħω* versus Re(*k*) and (**ii**) *ħ*Re(*ω*) versus *k* by using the COM model with simulated parameters (see supporting information) with varied MNW diameter (*D*) of (from right to left, colors from dark to light) 50, 75, 100, 150, 200, 300, 400 nm, respectively, showing less profound back-bending features and smaller *ħ*Ω_*R*_ with increasing diameters. Gray dashed lines: exciton resonance. **b** *ħ*Ω_*R*_ versus *D*. The numerically obtained result (squared green line) coincides well with one calculated using Eq. (5) (dotted pale green line). Despite the reduced *ħ*Ω_*R*_, the strong coupling criterion is fulfilled for every diameter presented compared to the overall damping of the coupled system (gray dashed line)

Besides the versatility in operation wavelengths, the PEPP and strong coupling behaviors can also be manipulated by the diameter (*D*) of the MNW (Fig. 5), exhibiting large tunability with tailored dispersions. As shown in Fig. 5a, by increasing *D* from 50 to 400 nm, the back-bending feature in the anomalous region becomes less profound (Fig. 5a(i)) with a smaller Rabi splitting (Fig. 5a(ii)) in dispersions. The corresponding *ħ*Ω_*R*_ varies from ~ 85.7 to ~ 34.2 meV (dotted line in Fig. 5b). To understand this decline trend, we derive an analytical expression of Ω_*R*_ in a general form, which is calculated via the coupling strength between the SPP and the exciton resonance as [46, 47]

2 $${\Omega_R} = 2g = {{2\mu \sqrt N {E_m}} \mathord{\left/ {\vphantom {{2\mu \sqrt N {E_m}} \hbar }} \right. \kern-\nulldelimiterspace} \hbar }$$

where *g* is the zero-detuning coupling coefficient, *µ* is the transition dipole moment of the exciton, *N* is the numbers of the excitons, and *E*_*m*_ is the electric field amplitude of the SPP per photon. Since the WS_2_ is described by the Lorentz oscillator model, the overall transition dipole moments term $\mu \sqrt N$ can be estimated as [23, 48]

3 $$\mu \sqrt N = \mu \sqrt {\rho V} = \sqrt {\hbar {\varepsilon_0}f\omega_p^2V/2{\omega_{ex}}} ,$$

where *ρ*, *fω*_*p*_^2^, *ω*_ex_ represent the oscillator density, oscillator strength, and resonance frequency of the WS_2_. *V* is the volume of the WS_2_ layer that can be obtained from its geometric thickness *t*_*l*_ as $V = \pi (D + {t_l}){t_l}L = {A_l}{L_m}$, where *A*_*l*_ denotes the cross-sectional area of the WS_2_ layer. On the other hand, *E*_*m*_ can be approximately calculated through the mode volume *V*_*m*_ [49]

4 $${E_m} = \sqrt {{{\hbar \omega } \mathord{\left/ {\vphantom {{\hbar \omega } {2{\varepsilon_0}{\varepsilon_b}{V_m}}}} \right. \kern-\nulldelimiterspace} {2{\varepsilon_0}{\varepsilon_b}{V_m}}}} = \sqrt {{{\hbar \omega } \mathord{\left/ {\vphantom {{\hbar \omega } {2{\varepsilon_0}{\varepsilon_b}{A_m}{L_m}}}} \right. \kern-\nulldelimiterspace} {2{\varepsilon_0}{\varepsilon_b}{A_m}{L_m}}}} ,$$

where *A*_*m*_ is the mode area of the SPP mode. At the zero-detuning where *ω* = *ω*_ex_, by substituting Eqs. (3–4) into Eq. (2), we can obtain the Ω_*R*_ as

5 $${\Omega_R} = \sqrt {{{f\omega_p^2{A_l}} \mathord{\left/ {\vphantom {{f\omega_p^2{A_l}} {{\varepsilon_b}{A_m}}}} \right. \kern-\nulldelimiterspace} {{\varepsilon_b}{A_m}}}} .$$

As shown by the pale green dotted line in Fig. 5b, *ħ*Ω_*R*_ obtained using Eq. (5) agrees reasonably well with the simulated one (green squared line), further validating our result. The decline trend in *ħ*Ω_*R*_ is due to the increasing *A*_*m*_ in a thicker MNW with a consequent weaker plasmon–exciton interaction. Despite of the reduced *ħ*Ω_*R*_, the strong coupling condition is still fulfilled for every diameter within the range we presented compared to the overall damping of the system (gray dashed line). On the other hand, although *ħ*Ω_*R*_ shown here (e.g., *ħ*Ω_*R*_/*ħω*_ex_ =  ~ 4.2% for *D* = 50 nm) cannot reach the ultra-strong coupling regime (*ħ*Ω_*R*_/*ħω*_ex_ > 20% [50]), it can be further enhanced by decreasing *A*_*m*_. And potential strategies for reducing *A*_*m*_ may include reducing the diameter of the MNW [32] and utilizing nano-focusing structures (e.g., tapered plasmonic waveguides) [51, 52]. Along with the tailored *ħ*Ω_*R*_ and dispersions, waveguiding properties of PEPPs can also be engineered with varied MNW diameters, exhibiting large tunability with *A*_*m*_ (~ 0.000169 µm^2^ to ~ 0.09 µm^2^) and *L*_*m*_ (~ 0.24 µm to ~ 60 µm) ranging across two orders of magnitudes, and FOM up to 250 (see Additional file 1: Fig. S4–S9 for waveguiding properties of MNW with *D* from 75 to 400 nm). Note that even for the thickest MNW (*D* = 400 nm) we discussed here, the energy can still be tightly confined within the ultra-thin WS_2_ layer at the 1-nm level due to the strong coupling (see Additional file 1: Fig. S10).

### Exceptional Confinement-Loss and Confinement-Momentum Trade-Offs

**Fig. 6:**
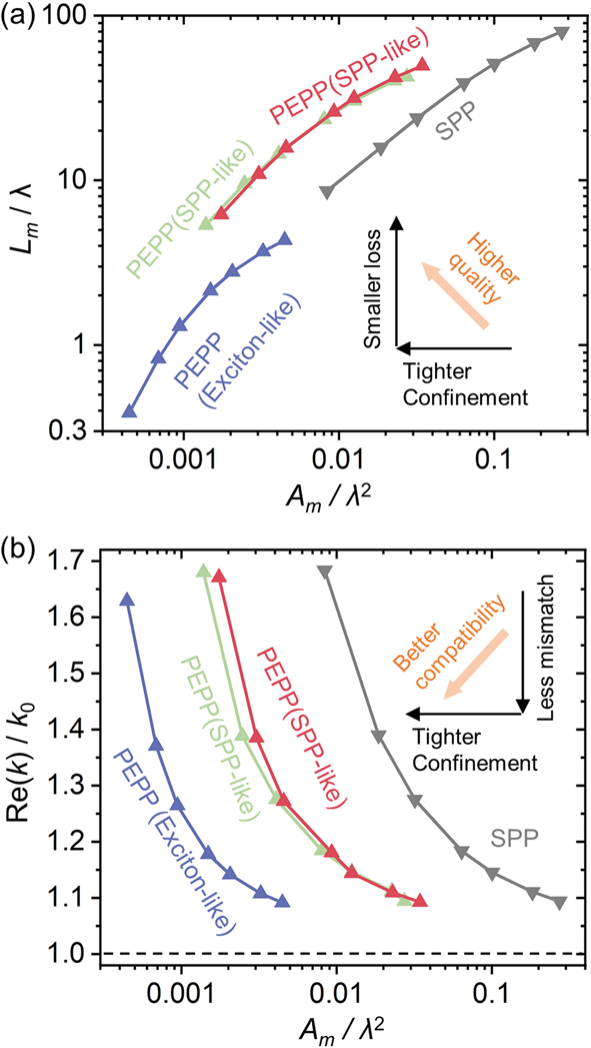
Comparison between PEPPs and SPPs in parametric plots. **a** Normalized propagation length (*L*_*m*_/*λ*) versus normalized mode area (*A*_*m*_/*λ*^2^), revealing the trade-off between confinement and loss. Inset: since the confinement/propagation length increases toward the left/up direction, the trajectory located at the upper-left area indicates the best trade-off relation. **b** Normalized momentum (Re(*k*)/*k*_0_, *k*_0_: wavevectors of free-space photons) versus normalized mode area (*A*_*m*_/*λ*^2^), revealing the trade-off between confinement and momentum mismatch to free-space photons (black dashed line). Inset: since the confinement/momentum mismatch increases toward the left/up direction, trajectories located at the bottom-left area indicate the best trade-off relation. Blue lines with triangles: exciton-like PEPPs for maximum confinement. Green and red lines with triangles: SPP-like PEPPs for maximum FOMs. Gray lines with triangles: SPPs

In this section, we show that the PEPP provided by our strongly coupled MNW-WS_2_ structure represents another kind of waveguiding polaritons that is superior than the original SPP in MNWs. To understand its merits, parametric plots allowing direct comparison between different polaritons [53] are provided in Fig. 6. Due to two types of mode characters for the PEPP, operations in the exciton-like region at the maximum confinement wavelength (e.g., blue star in Fig. 4c for *D* = 50 nm) and in the SPP-like region with the two local maximum FOMs (e.g., green square and diamond in Fig. 4c for *D* = 50 nm) are considered. Figure 6a gives parametric plots of normalized propagation length (*L*_*m*_/*λ*) versus normalized mode area (*A*_*m*_/*λ*^2^), showing the confinement-loss trajectory over the range of *D* from 50 to 400 nm. As is shown, polaritons of the same character type follows the same trajectory, allowing a fair comparison between the PEPP and the SPP that is independent of the geometric size. As indicated by the inset, the trajectories toward the upper-left area indicate the best trade-off between confinement and loss. Result shows that although the exciton-like PEPP follows the similar trajectory line with the SPP, it can reach an ultra-deep subwavelength region (< 0.005 *λ*^2^) that is challenging for the SPP, while for SPP-like PEPPs, they exhibit distinct trajectories from the SPP, offering higher qualities with a more efficient confinement-loss relation.

Besides the trade-off between confinement and loss, another fundamental hurdle in plasmonics is the trade-off between confinement and momentum [36]. For the SPP, the momentum *ħ*Re(*k*) is always larger than the momentum of the free-space photon *ħk*_0_ (*k*_0_ is the wavevector in vacuum), resulting in momentum mismatch that needs to be compensated for optical excitation [33]. However, the tight confinement is usually achieved at the cost of a large momentum mismatch to the photon, which hinders the efficient SPP excitation and may further limits its application (e.g., ultra-thin MNW) [35]. Figure 6b gives parametric plots of normalized momentum (Re(*k*)/*k*_0_) vs. normalized mode area (*A*_*m*_/*λ*), where the trajectory towards the bottom-left area represents the best performance in confinement-momentum relations. As is shown, PEPPs outperform the SPP and offer the capability to realize a much tighter confinement with a smaller momentum mismatch to the free-space photon (e.g., for the SPP, *A*_*m*_ of ~ 0.01 *λ*^2^ with a Re(*k*)/*k*_0_ of ~ 1.62, while for PEPPs, *A*_*m*_ of ~ 0.01 *λ*^2^ (SPP-like) and ~ 0.0015 *λ*^2^ (exciton-like) can be achieved at a Re(*k*)/*k*_0_ as small as ~ 1.17). The smaller momentum mismatch indicates the less momentum needs to be compensated, which may facilitate a more efficient polariton excitation [36] with an improved compatibility for integrated photonic/plasmonic structures. Such compatibility offers the opportunity to realize high-performance hybrid polaritonic components and devices (e.g., by integrating with low-loss photonic waveguides), where ultra-deep subwavelength confinement and low propagation loss can be simultaneously achieved.

### Considerations for Practical Applications

**Fig. 7:**
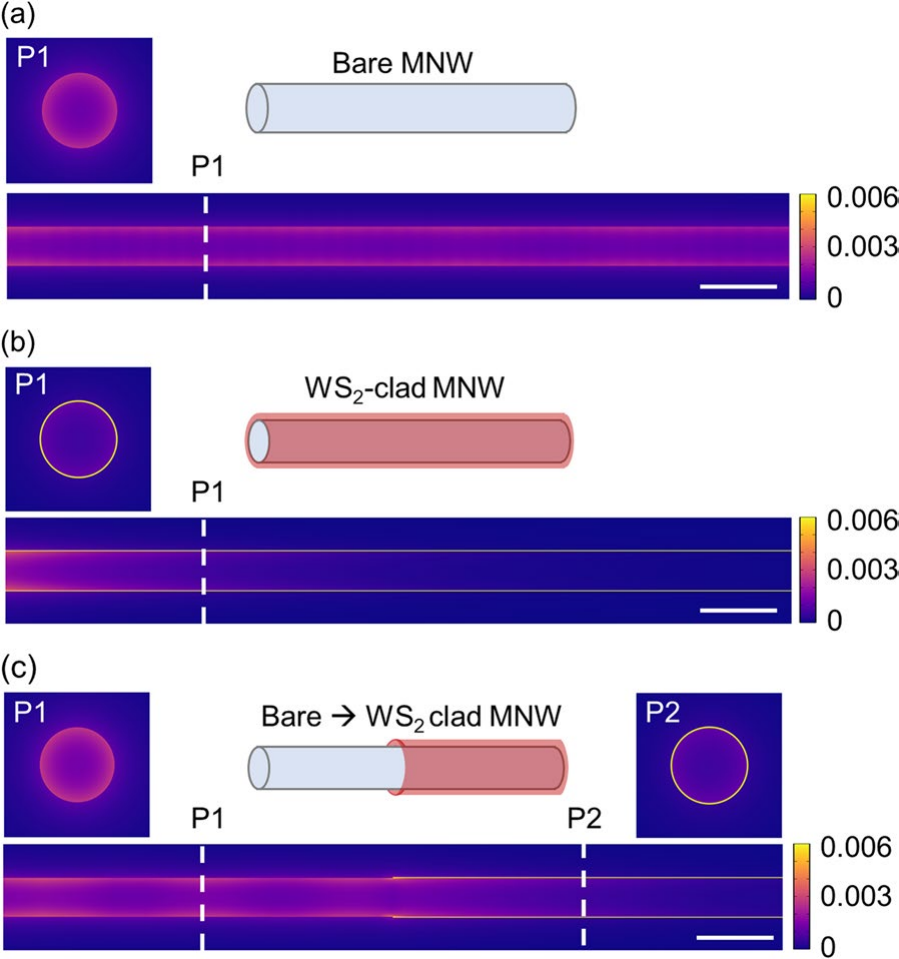
Energy density distributions at the transverse cross section plane and along the propagation direction of **a** a bare MNW without cladding, **b** a WS_2_-clad MNW, and **c** coupling from a bare MNW to a WS_2_-clad MNW at the excitonic resonance based on 3D simulations. For better visualization, energy densities are normalized and plotted in a color bar with saturation (for more information, see Additional file 1: Fig. S12). P1 and P2 represent the transverse cross section planes located at the positions indicated by the white dashed lines. Scale bars (right bottom white lines): 100 nm

The fabrication of the proposed structure is experimentally possible and can be realized by various techniques for the integration of nano-waveguides and 2D materials [54–56]. For instance, a bare Ag MNW with a uniform diameter and smooth surface can be chemically synthesized by a solution [57]- or vapor-phase [58] method. The monolayer 2D material can be wrapped around the MNW via micro-manipulation under an optical microscope [54, 55] or a capillary-force-driven rolling-up process [56]. By selectively wrapping the WS_2_ monolayer on one segment of the MNW, we can seamlessly integrate our proposed WS_2_-clad MNW to the bare MNW for efficient external coupling. For demonstration, 3D simulations are performed (see Additional file 1: Fig. S11 for configurations). Energy density distributions of a bare MNW without cladding (Fig. 7a), a WS_2_-clad MNW (Fig. 7b), and the integrated structure (Fig. 7c) are, respectively, provided. Since the energy is highly concentrated in the 1-nm WS_2_ cladding, energy densities are normalized and plotted in a color bar with saturation [59] for better visualization and comparison. As is illustrated by the schematic plot in Fig. 7c, the left part of the integrated structure is the bare MNW without cladding, while only the right part is wrapped with the WS_2_ layer. In this case, the plasmon mode of the bare part (inset P1 in Fig. 7c) is firstly excited and then efficiently converted to the PEPP mode (inset P2 in Fig. 7c) at the right part. Note that the simulated mode profiles for the plasmon and PEPP modes (insets P1 and P2 in Fig. 7c) in the integrated structure also agree well with the one individually obtained in the bare MNW (inset P1 in Fig. 7a) and WS_2_-clad MNW (inset P1 in Fig. 7b). The advantage of this external coupling strategy is that the excitation techniques of the plasmon mode in the bare MNW are very mature and have been extensively investigated (e.g., prism coupling, lens-focusing coupling, direct nanowire-to-nanowire coupling, and emitter coupling) [33, 35]. Hence, we only need to focus on the coupling efficiency (*η*_ext_) from the bare MNW part to the WS_2_-clad MNW part, which can be calculated as:

6 $${\eta_{{\text{ext}}}} = {(\left| {{S_{21}}} \right|/\exp (\operatorname{Im} ({k_1}){L_1} + \operatorname{Im} ({k_2}){L_2}))^2},$$

where *k*_1_ (*k*_2_) and *L*_1_ (*L*_2_) are the propagation constant and the length of the bare (WS_2_-clad) MNW part in Fig. 7c. *S*_21_ is the transmission *S* parameter obtained from the 3D simulation (see Sect. 4 in supporting information for more details about 3D simulation). The calculated *η*_ext_ is ~ 90%, indicating an efficient coupling from the bare MNW to our proposed WS_2_-clad MNW. It is worth mentioning that for polarizations of the excitation mode in MNWs, although the fundamental TM and second-order HE modes do not have cutoffs, we only focus on the fundamental TM mode under the following considerations: (1) The single-mode operation is usually favorable [32, 33] and can be readily realized for practical applications (e.g., by aligning the polarization of the incident light to the long axis of the MNW to only excite the TM mode) [35, 39]; (2) more importantly, the HE mode has a dramatically increasing *A*_*m*_ with the decreasing MNW diameter, making it non-confined with an almost infinitely large *A*_*m*_ at the small diameter we discussed here [35], which is difficult to be excited and not suitable for strong coupling applications.

**Fig. 8:**
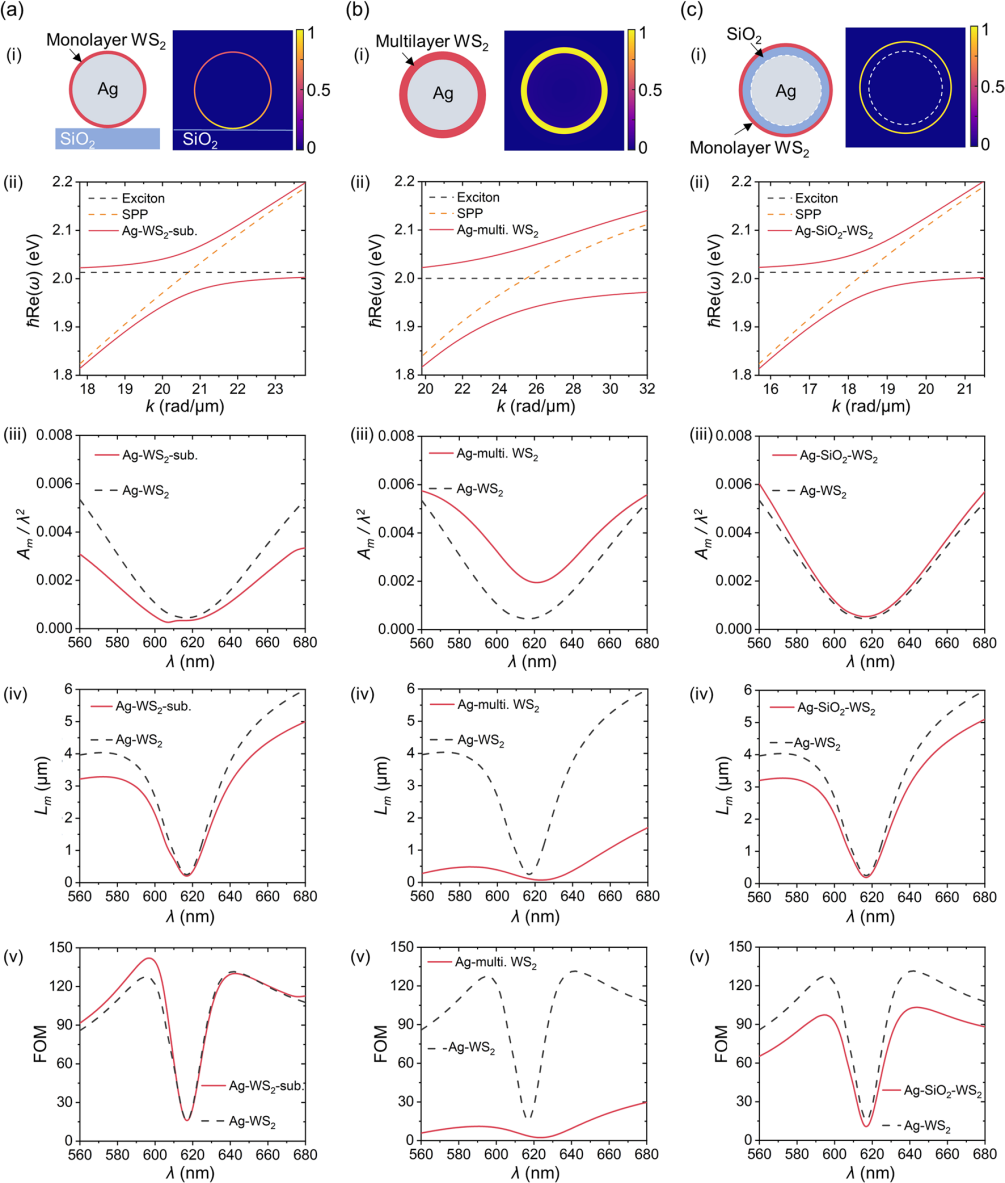
Typical situations of **a** a silica substrate-supported WS_2_-clad MNW (Ag-WS_2_-sub.), **b** a multilayer WS_2_-clad MNW (Ag-multi. WS_2_), and **c** a WS_2_-clad MNW with a silica insulating layer (Ag-SiO_2_-WS_2_). (**i**) Schematic plot (left) and mode profile at the excitonic resonance (right). (**ii**) Rabi splitting dispersion of the corresponding PEPPs (red lines). Yellow and black dashed lines: SPPs and excitons. (**iii**) Normalized mode area *A*_*m*_/*λ*^2^. (**iv**) Propagation length *L*_*m*_. (**v**) Figure of merit (FOM). The free-standing WS_2_-clad MNW (Ag-WS_2_) is also provided for comparison and plotted as black dashed lines in (**ii**–**v**). The thickness of the insulating layer in **c** is 5 nm. The diameter of the Ag MNW here is 50 nm

Finally, for guiding practical applications, we investigate three typical situations including a substrate-supported WS_2_-clad MNW, a multilayer WS_2_-clad MNW, and a WS_2_-clad MNW with an insulating layer between the metal and WS_2_, which are shown, respectively, in Fig. 8a–c.

For the substrate-supported case (Fig. 8a), we calculate the situation of a WS_2_-clad MNW on a silica substrate (*n* = 1.45). As is shown, the energy can be well concentrated within the WS_2_ layer (Fig. 8a(i)), and the strong coupling effect is still valid at the presence of the substrate, exhibiting a similar Rabi energy (*ħ*Ω_*R*_ =  ~ 86.9 meV) compared to the free-standing case (*ħ*Ω_*R*_ =  ~ 85.7 meV) (Fig. 8a(ii)). For the waveguiding properties, the substrate-supported MNW features asymmetric SPP mode with improved waveguiding properties [32]. Since the PEPP consists of both SPP and exciton, the mode profile of the PEPP also becomes asymmetric with more energy distributed towards the substrate side (Fig. 8a(i)). Meanwhile, compared to the symmetric PEPP mode in the free-standing WS_2_-clad Ag nanowire (black dashed lines in Fig. 8a(iii–v)), the asymmetric PEPP mode has a tighter confinement (red line in Fig. 8a(iii)), a slightly shorter *L*_*m*_ (red line in Fig. 8a(iv)), and an overall enhancement in FOM (red line in Fig. 8a(v)), which may be mainly due to the improved properties of the asymmetric SPP [45, 60, 61]. As can be seen, at the wavelengths far away from the excitonic resonance, PEPPs are mostly composed of SPPs, resulting in a relatively large difference in the waveguiding properties between the free-standing and the substrate-supported cases (e.g., Fig. 8a(iii), (iv)), while at the wavelengths close to the excitonic resonance, such difference becomes almost negligible since the excitons contribute mostly to the PEPPs.

For the multilayer case, a Ag MNW with a multilayer WS_2_ cladding is investigated and schematically illustrated in Fig. 8b(i). The thickness (*t*_*l*_ = 4 nm) and permittivity parameters (*ε*_*b*_ = 20.25, *ħ*^*2*^*fω*_*p*_^2^ = 0.8 eV^2^, *ħω*_ex_ = 2 eV and *ħγ*_ex_ = 50 meV) of the multilayer WS_2_ are taken from Ref. [62]. Due to the larger overall transition dipole moments ($\mu \sqrt N$ which is proportional to the thickness *t*_*l*_, see Eq. 3), the Rabi energy (*ħ*Ω_*R*_ =  ~ 127.5 meV) of the strong coupling is greater than the monolayer case (Fig. 8b(ii)). On the other hand, compared to the monolayer case, the increase in the *t*_*l*_ is not favorable for the energy confinement to achieve a small *A*_*m*_ (Fig. 8b(iii)). Moreover, the exciton damping *ħγ*_*ex*_ of the multilayer WS_2_ (50 meV) is much larger than that of the monolayer WS_2_ (22 meV), leading to extra loss in the coupling system. As a result, compared to the monolayer WS_2_-clad Ag MNW (black dashed lines in Fig. 8b(iv, v), PEPP modes in the multilayer WS_2_-clad Ag MNW (red lines in Fig. 8b(iv, v)) exhibit much shorter *L*_*m*_ and much poorer FOM, which may limit their waveguiding applications.

In some applications, the direct contact of metal and TMD materials may induce weak electronic coupling that affects the exciton formation in the WS_2_ and the electron dynamics in the metal [63, 64]. To minimize the influence, one may use the structure of a core/shell MNW with a TMD layer. As is schematically illustrated in Fig. 8c(i), a Ag-core/silica-shell MNW [65, 66] with a monolayer WS_2_ cladding is used, where the silica shell serves as an insulating layer between the Ag and the WS_2_. For such configuration, the strong coupling can also be achieved (Fig. 8c(ii)) with comparable waveguiding properties to the ones without the dielectric insulating layer (Fig. 8c(iii–v)).

## Conclusion

In summary, we have theoretically demonstrated plasmon–exciton strong couplings in a single Ag MNW with a monolayer WS_2_ cladding, generating PEPPs with exceptional properties. As to strong coupling behaviors, solutions in both complex-momentum and complex-frequency planes have been investigated, revealing the back-bending and anti-crossing features with tunable Rabi splitting energies that can be controlled by varying the diameter of MNWs. We have also shown that results obtained from numerical simulations exhibit very good agreement to the ones obtained from the COM model and the analytical estimation. For the generated PEPPs, fractions, model profiles, energy density distributions, waveguiding properties including mode areas, propagation lengths, and FOMs have been investigated to provide a comprehensive characterization. We have shown that energy distributions can be reformed by the strong coupling, yielding a much tighter confinement of the PEPP than the original SPP. Meanwhile, due to the hybrid nature of polaritons, the PEPP is highly versatile possessing SPP-like and exciton-like characters that can be operated at different wavelengths. When operated at the exciton-like region, the PEPP can exert its full potential to reach the ultra-deep subwavelength confinement, while in the SPP-like region, the PEPP exhibits excellent FOM with long propagation distance and tight confinement. Moreover, by comparing trajectories in the parametric plots, we have also demonstrated that PEPPs represent another kind of waveguiding polaritons with exceptional confinement-loss and confinement-momentum trade-offs that outperform the SPP in MNW. Such exceptional properties are favorable for integrations with low-loss photonic waveguides to form hybrid photonic-polaritonic structures, making it possible to bypass the barriers of nano-plasmonics with simultaneous realization of ultra-deep subwavelength confinement and low propagation loss. Note that this strong coupling scheme can be extended to other configurations of different nano-waveguides and TMDs (e.g., a TMD-clad pentagonal MNW and a MNW on a flat TMD, see Additional file 1: Figs. S13–S14), offering a simple and promising guided-wave platform to manipulate the plasmon–exciton interaction at the ultra-deep subwavelength scale. The generated PEPPs with exceptional properties may open new opportunities for various integrated polaritonic components and devices such as on-chip polaritonic circuits, polariton lasers, and all-optical switches.

## Supplementary Information

**Additional file 1:** Supporting information including numerical methods and equations for characterization, parameters for the coupled-oscillator (COM) model, waveguiding properties of PEPP with varied MNW diameters, 3D simulations, and extending the strong coupling strategy to other structures.

## Data Availability

The datasets used and/or analyzed during the current study are available from the corresponding author on reasonable request.

## Abbreviations

TMD: Transition metal dichalcogenides
PEPPs: Propagating exciton–plasmon polaritons
SPPs: Surface plasmon polaritons
1D: One dimension
2D: Two dimensions
Ag: Silver
WS_2_: Tungsten disulfide
FWHM: Full width at half maximum
MNWs: Metal nanowires
COM: Coupled-oscillator model
FOM: Figure of merit
