# Supplementary material for: Ultra-confined Propagating Exciton–Plasmon Polaritons Enabled by Cavity-Free Strong Coupling: Beating Plasmonic Trade-Offs
Source: Nanoscale Res Lett. 2022 Nov 18;17:109. doi: 10.1186/s11671-022-03748-7 (PMC9674826; doi:10.1186/s11671-022-03748-7)
Supplement: Supplementary file 1 — Additional file 1: Supporting information including numerical methods and equations for characterization, parameters for the coupled-oscillator (COM) model, waveguiding properties of PEPP with varied MNW diameters, 3D simulations, and extending the strong coupling strategy to other structures. [file 11671_2022_3748_MOESM1_ESM.pdf]

Supporting Information for

Ultra-confined propagating exciton-plasmon polaritons enabled by cavity-free strong coupling: beating plasmonic trade-offs

Yipei Wang[1]*, Aoning Luo[1], Chunyan Zhu[1], Zhiyong Li[2, 3, 4], and Xiaoqin Wu[1]*

[1]Key Laboratory of Optoelectronic Technology & Systems (Ministry of Education), College of Optoelectronic Engineering, Chongqing University, Chongqing 400044, People's Republic of China

[2]State Key Laboratory of Modern Optical Instrumentation, College of Optical Science and Engineering, Zhejiang University, Hangzhou 310027, People's Republic of China

[3]Jiaxing Key Laboratory of Photonic Sensing & Intelligent Imaging, Jiaxing 314000, People's Republic of China

[4]Jiaxing Intelligent Optics & Photonics Research Center, Jiaxing Research Institute Zhejiang University, Jiaxing 314000, People's Republic of China

*wangyp@cqu.edu.cn; xiaoqinwu@cqu.edu.cn

1. Numerical methods and equations for characterization

The permittivity of the monolayer WS_2 is described by a Lorentz oscillator model: $\varepsilon_{WS2} = \varepsilon_b + f\omega_p^2 / (\omega_{ex}^2 - \omega^2 - i\gamma_{ex}\omega)$ with parameters from Ref. [1], where the non-resonant background permittivity ε_b = 16, the oscillator strength $\hbar^2 f\omega_p^2$ =1.85 eV^2, the resonant energy $\hbar\omega_{ex}$ = 2.013 eV and the damping (linewidth) $\hbar\gamma_{ex}$=22 meV. The permittivity of the Ag nanowire is described by an effective Drude model: $\varepsilon_{Ag} = \varepsilon_{b,Ag} - \omega_{p,Ag}^2 / (\omega^2 + i\gamma_{Ag}\omega)$ with parameters from Ref. [2], where $\varepsilon_{b,Ag}$=3.7, $\hbar\omega_{p,Ag}$=8.9 eV and $\hbar\gamma_{Ag}$=20 meV.

For the PEPP in our coupling system, it can be obtained by numerically solving the eigenvalue problem of the wave equation $(\nabla^2 - \varepsilon\mu \frac{\partial^2}{\partial t^2})E = 0$ with the time-harmonic field given by $E(x, y, z, t) = E(x, y)e^{i(kz-\omega t)}$, where μ and ε are permeability and permittivity, (x, y, z) represents the spatial coordinates indicated in Fig. 1, ω and k are the angular frequency and the wavevector parallel with the MNW, respectively. The solutions can either be complex ω with real k or complex k with real ω [3]. For the complex-ω solution, the eigenvalue of ω is solved by feeding the eigenequation with a real-valued k, where the real part (Re(ω)) and the imaginary part (Im(ω)) of the ω represent the eigenfrequency and the temporal damping of the PEPP, respectively. As to the complex-k solution, the eigenvalue of k is solved by feeding in a real-value ω, where the real part (Re(k)) and the imaginary part (Im(k)) of the k correspond to the propagation constant and the spatial damping along the propagation direction. The above eigenvalue problems are solved by using a finite element method (FEM) implemented by the

COMSOL Multiphysics. For the FEM calculation, the Wave Optics module in the COMSOL Multiphysics is used. The simulation region is discretized into a triangular mesh with a minimum element size of 0.05 nm, and terminated by perfectly matched layer (PML) boundary conditions. Similarly, the bare SPP mode without coupling to the exciton is calculated by setting the permittivity of the WS_2 to be its non-resonant background permittivity with the oscillator strength being zero (i.e. $\varepsilon_{WS2} = \varepsilon_b = 16$).

For characterization of the energy distribution, the fractional energy inside the MNW (η_m) and the WS_2 layer (η_l) are calculated as

$$\eta_{m,\,l} = \frac{\iint_{m,\,l} W(x,y)dxdy}{\iint_{tot} W(x,y)dxdy}, \tag{S1}$$

where the subscript indicates the integration area (m=MNW, l=WS_2, tot=total region) and $W(x, y)$ is the energy density. And for the dispersive and absorptive material, the energy density is calculated by $W(x,y) = \left[\varepsilon_0(\mathrm{Re}(\varepsilon) + 2\omega \mathrm{Im}(\varepsilon)/\gamma)|E(x,y)|^2 + \mu_0 |H(x,y)|^2\right]/4$ via the real part $\mathrm{Re}(\varepsilon)$, imaginary part $\mathrm{Im}(\varepsilon)$, and damping frequency γ of the complex permittivity [4].

For characterization of the spatial confinement and loss relation, the mode area (A_m) is defined as [5]

$$A_m = \frac{\iint_{tot} W(x,y)dxdy}{\max[W(x,y)]}. \tag{S2}$$

And the loss (damping along the z direction) is inversely proportional to the propagation length (L_m), which is calculated through the imaginary part of the complex-valued k [6]

$$L_m = \frac{1}{2\,\mathrm{Im}(k)}. \tag{S3}$$

2. Parameters for the coupled-oscillator (COM) model

The PEPP can be regarded as the hybridization of SPPs and excitons. In COM model, the eigen frequency of the PEPP can be obtained via the diagonalization of the Hamiltonian [7]

$$H = \hbar \begin{pmatrix} \omega_{SPP} - i\frac{\gamma_{SPP}}{2} & \frac{\Omega_R}{2} \\ \frac{\Omega_R}{2} & \omega_{ex} - i\frac{\gamma_{ex}}{2} \end{pmatrix}. \tag{S4}$$

Here $\hbar\omega_{ex}$=2.013 eV and $\hbar\gamma_{ex}$=22 meV are the exciton resonance energy and damping of the WS_2 material from the Lorentz model [1]. ω_{SPP}=Re(ω) and γ_{SPP}=2|Im(ω)| are the eigen frequency and damping frequency of the SPP mode, which are obtained from the complex-ω solution of the SPP mode. $\hbar\Omega_R/2$ represents the coupling strength at the zero detuning ($\omega_{SPP}=\omega_{ex}$).

For the parameters used in Fig. 5a(ii) in the main text, Figs. S1-S2 give the numerically obtained $\hbar\omega_{SPP}$ and $\hbar\gamma_{SPP}$ of the SPP (using the method in section1) with the varied MNW diameters (D) from 75 to 400 nm. For reference, the corresponding exciton counterparts $\hbar\omega_{ex}$ and $\hbar\gamma_{ex}$ are also plotted as black lines.

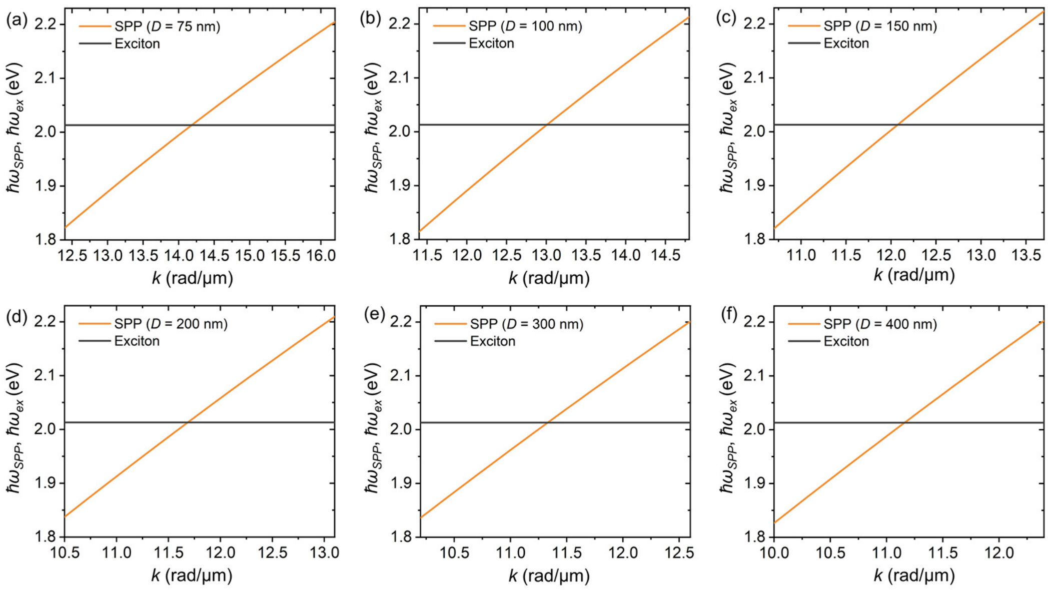

Fig. S1. $\hbar\omega_{SPP}$ and $\hbar\omega_{ex}$ of the SPPs (yellow lines) and excitons (black lines) with varied MNW diameters (D). (a) D = 75 nm, (b) D = 100 nm, (c) D = 150 nm, (d) D = 200 nm, (e) D = 300 nm, (f) D = 400 nm.

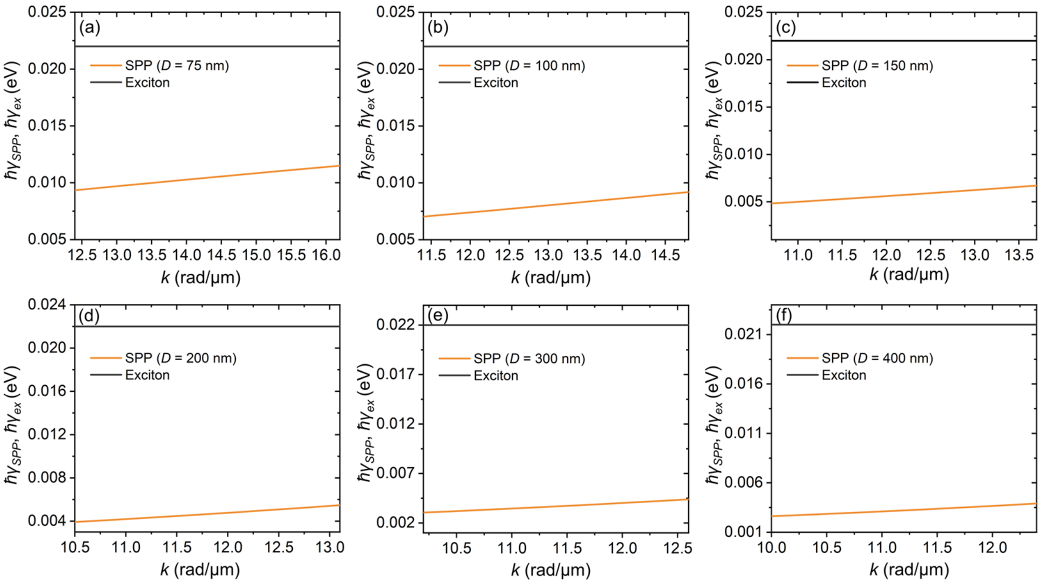

Fig. S2. $\hbar\gamma_{SPP}$ and $\hbar\gamma_{ex}$ of the SPPs (yellow lines) and excitons (black lines) with varied MNW diameters (D). (a) D = 75 nm, (b) D = 100 nm, (c) D = 150 nm, (d) D = 200 nm, (e) D = 300 nm, (f) D = 400 nm.

From the Fig. S1, we can also obtain the k at zero detuning (the cross point of the orange and black line where $\omega_{SPP} = \omega_{ex}$). By feeding the k at the zero detuning into the eigenequation for the PEPPs, we can obtain the eigenvalues of the upper polaritons and lower polaritons, yielding $\hbar\Omega_R$ listed in the Table S1.

Table S1. $\hbar\Omega_R$ for different diameters

	D=75 nm	D=100 nm	D=150 nm	D=200 nm	D=300 nm	D=400 nm
$\hbar\Omega_R$ (meV)	68.73	53.58	45.41	41.09	36.60	34.18

With the parameters shown in Figs. S1-S2 and Table S1, the dispersion relation in terms of $\hbar\text{Re}(\omega)$ vs. k of the PEPP for MNWs with D from 75 to 400 nm in Fig. 5a(ii) can be obtained

using the COM model. For verification, Fig. S3 gives the comparison with $D = 100$ nm between the one obtained using COM model and the one from the simulation, showing excellent agreement with each other.

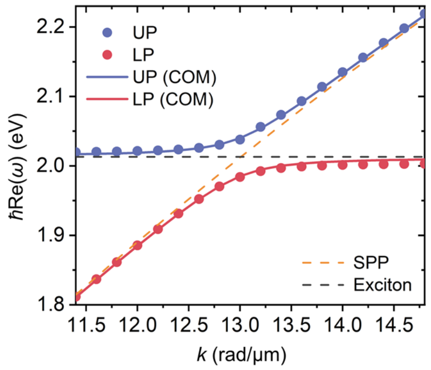

Fig. S3. Dispersion relation in terms of $\hbar\mathrm{Re}(\omega)$ vs. k. The numerically obtained result for the PEPP is denoted by blue (upper branch, UP) and red dots (lower branch, LP), showing excellent agreement to the result obtained from the coupled-oscillator model (COM, blue and red lines). Yellow and black dashed lines: SPP and excitons.

3. Waveguiding properties of PEPP with varied MNW diameters

Figs. S4-S9 give the calculated waveguiding properties including Re(k), Im(k), mode area (A_m), propagation length (L_m), and figure-of-merit (FOM) of the PEPP with varied diameters (D) of MNWs, showing large tunability with respect to the wavelength (λ) and the diameters.

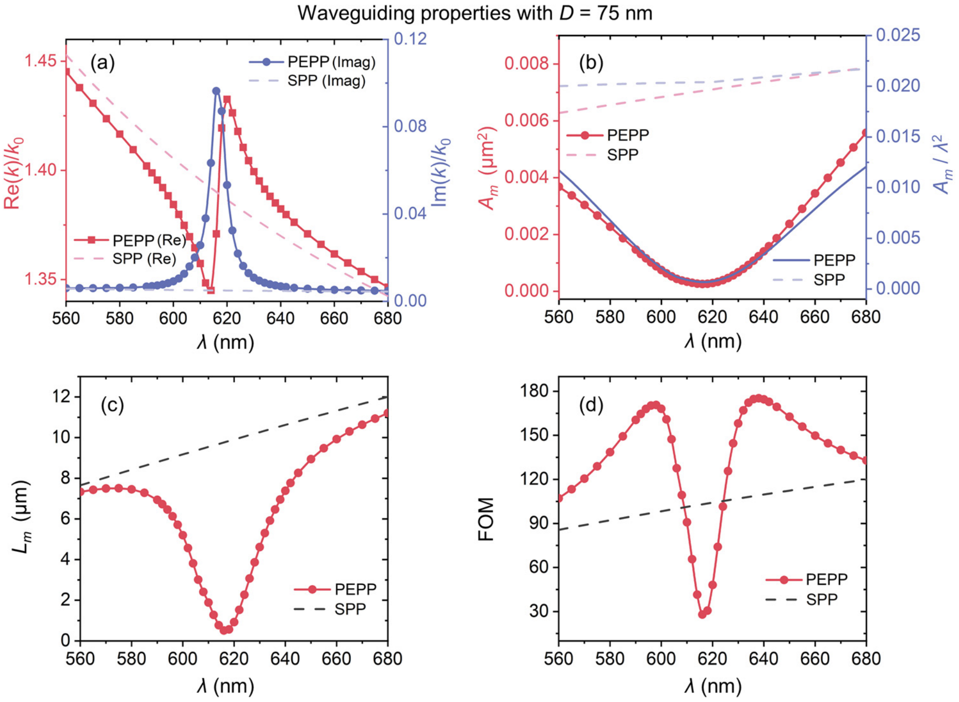

Fig. S4. Waveguiding properties of the PEPP with a MNW diameter of 75 nm. (a) Normalized $\mathrm{Re}(k)/k_0$ (left y axis) and $\mathrm{Im}(k)/k_0$ (right y axis), where k_0 is the free-space wavevector. Red squared and dashed lines denote $\mathrm{Re}(k)/k_0$ of the PEPP and SPP. Blue dotted and dashed line denote $\mathrm{Im}(k)/k_0$ of the PEPP and SPP. (b) Mode area A_m (left y axis) and normalized mode area A_m/λ^2 (right y axis). (c) Propagation lengths

L_m. (d) Figure of merit FOM. Red dotted and black dashed lines in (b-d): PEPP and SPP.

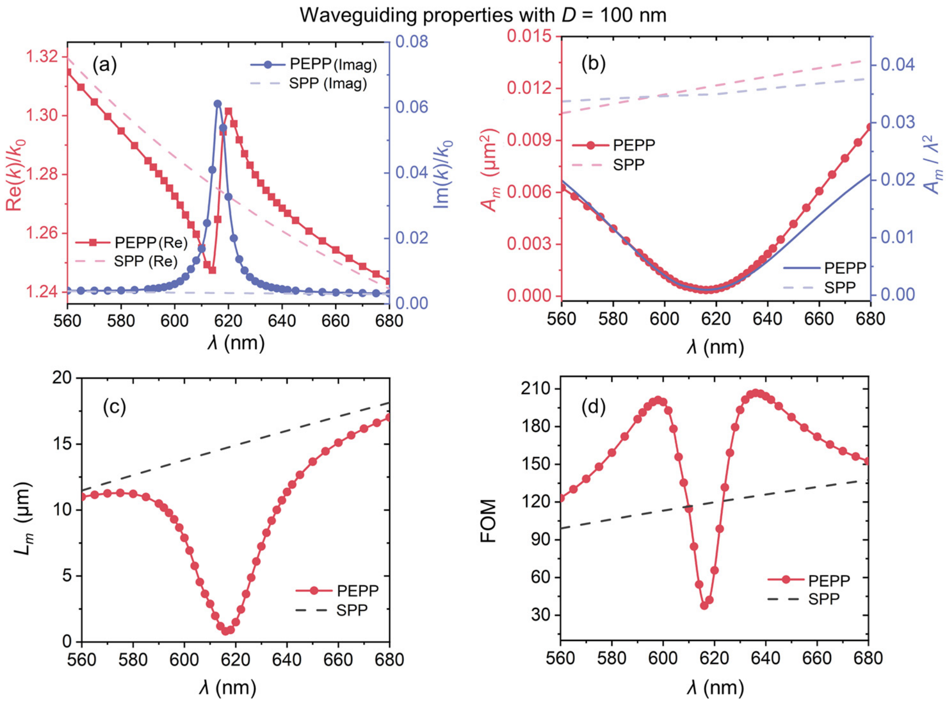

Fig. S5. Waveguiding properties of the PEPP with a MNW diameter of 100 nm. (a) Normalized Re(k)/k_0 (left y axis) and Im(k)/k_0 (right y axis), where k_0 is the free-space wavevector. Red squared and dashed lines denote Re(k)/k_0 of the PEPP and SPP. Blue dotted and dashed line denote Im(k)/k_0 of the PEPP and SPP. (b) Mode area A_m (left y axis) and normalized mode area A_m/λ^2 (right y axis). (c) Propagation lengths L_m. (d) Figure of merit FOM. Red dotted and black dashed lines in (b-d): PEPP and SPP.

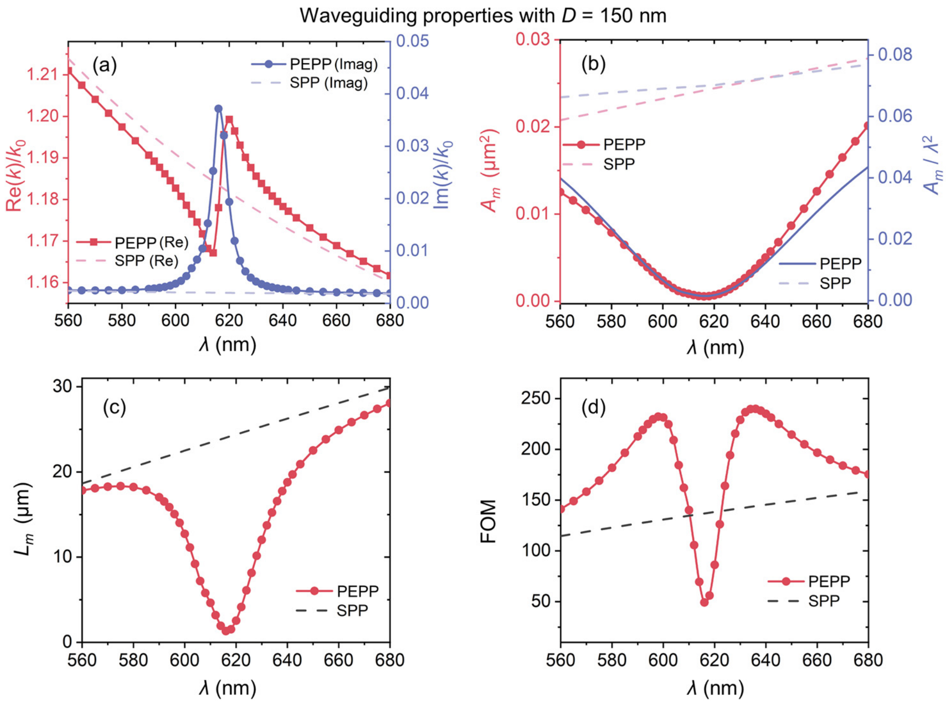

Fig. S6. Waveguiding properties of the PEPP with a MNW diameter of 150 nm. (a) Normalized Re(k)/k_0 (left y axis) and Im(k)/k_0 (right y axis), where k_0 is the free-space wavevector. Red squared and dashed

lines denote $\mathrm{Re}(k)/k_0$ of the PEPP and SPP. Blue dotted and dashed line denote $\mathrm{Im}(k)/k_0$ of the PEPP and SPP. (b) Mode area A_m (left y axis) and normalized mode area A_m/λ^2 (right y axis). (c) Propagation lengths L_m. (d) Figure of merit FOM. Red dotted and black dashed lines in (b-d): PEPP and SPP.

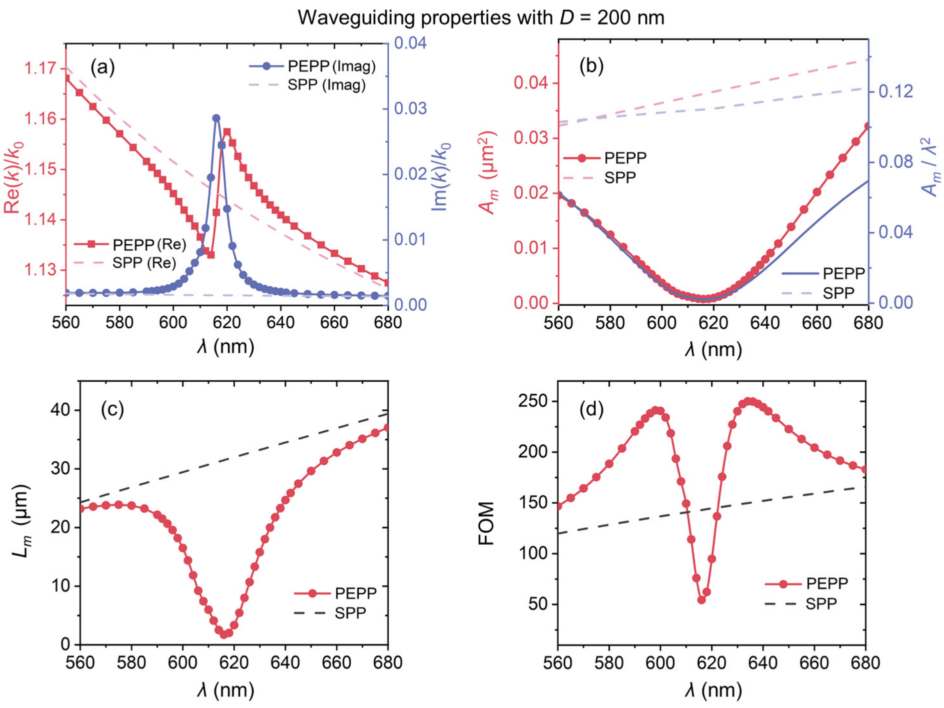

Fig. S7. Waveguiding properties of the PEPP with a MNW diameter of 200 nm. (a) Normalized $\mathrm{Re}(k)/k_0$ (left y axis) and $\mathrm{Im}(k)/k_0$ (right y axis), where k_0 is the free-space wavevector. Red squared and dashed lines denote $\mathrm{Re}(k)/k_0$ of the PEPP and SPP. Blue dotted and dashed line denote $\mathrm{Im}(k)/k_0$ of the PEPP and SPP. (b) Mode area A_m (left y axis) and normalized mode area A_m/λ^2 (right y axis). (c) Propagation lengths L_m. (d) Figure of merit FOM. Red dotted and black dashed lines in (b-d): PEPP and SPP.

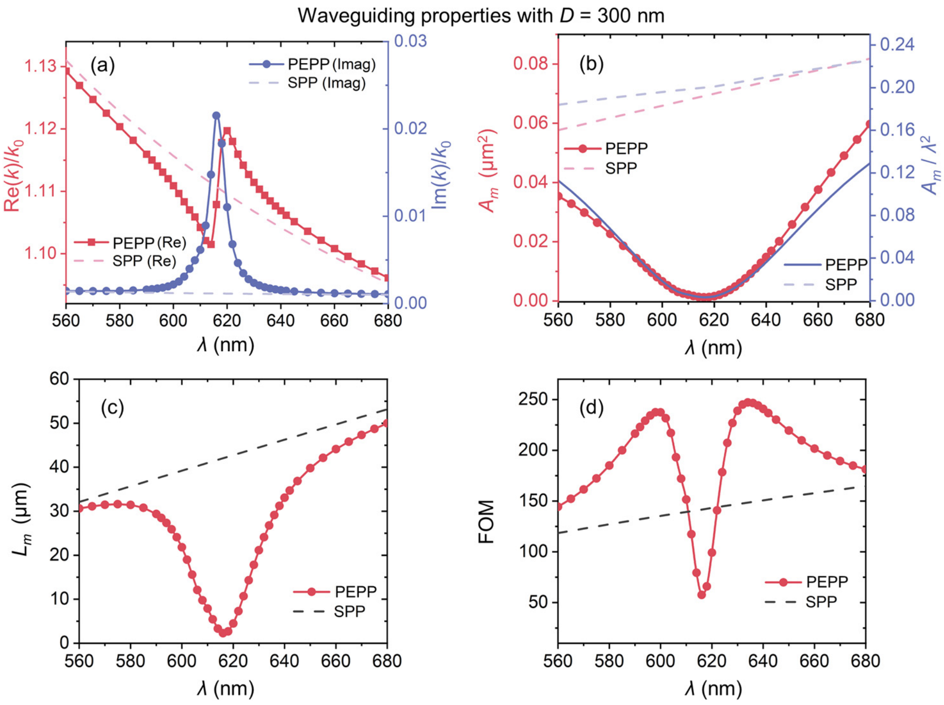

Fig. S8. Waveguiding properties of the PEPP with a MNW diameter of 300 nm. (a) Normalized Re(k)/k_0 (left y axis) and Im(k)/k_0 (right y axis), where k_0 is the free-space wavevector. Red squared and dashed lines denote Re(k)/k_0 of the PEPP and SPP. Blue dotted and dashed line denote Im(k)/k_0 of the PEPP and SPP. (b) Mode area A_m (left y axis) and normalized mode area A_m/λ^2 (right y axis). (c) Propagation lengths L_m. (d) Figure of merit FOM. Red dotted and black dashed lines in (b-d): PEPP and SPP.

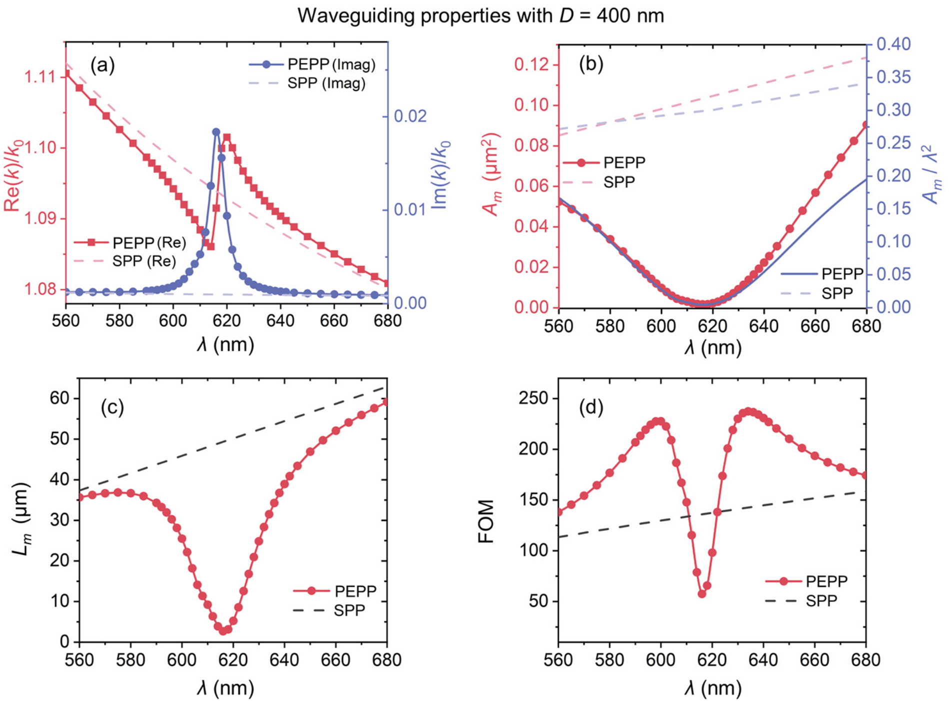

Fig. S9. Waveguiding properties of the PEPP with a MNW diameter of 400 nm. (a) Normalized Re(k)/k_0 (left y axis) and Im(k)/k_0 (right y axis), where k_0 is the free-space wavevector. Red squared and dashed lines denote Re(k)/k_0 of the PEPP and SPP. Blue dotted and dashed line denote Im(k)/k_0 of the PEPP and SPP. (b) Mode area A_m (left y axis) and normalized mode area A_m/λ^2 (right y axis). (c) Propagation lengths L_m. (d) Figure of merit FOM. Red dotted and black dashed lines in (b-d): PEPP and SPP.

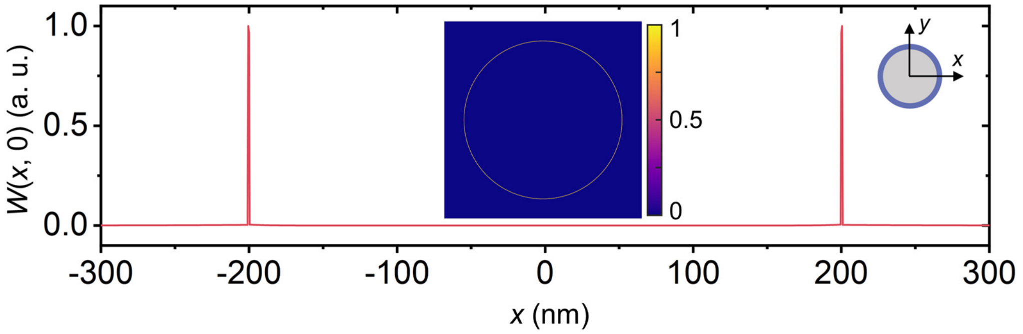

Fig. S10. Normalized energy density along x direction $W(x, 0)$ for a 400-nm-diameter MNW at λ=616 nm. The measured full width at half maximum (FWHM) of the peak is ~1 nm. Inset (middle): mode profiles indicating the energy tightly confined within the WS_2 layer. Inset (right): coordinates configuration on the cross section.

4. 3D simulations

For 3D simulations, the geometrical configuration with Cartesian coordinates is shown in

Fig. S11, where a MNW with the cross section in the *x*-*y* plane is placed along the *z* axis (propagation direction). The simulation region is firstly discretized into a triangular mesh with a minimum element size of 0.2 nm at the transverse cross section, and swept along the propagation direction. The length of the MNW (*L*) is set to be 1 μm with two numeric ports [8] deployed at the left-side- and right-side-boundaries. The left port is used as an input for exciting the waveguide, while the right one is used as an exit to detect and absorb the outgoing wave. For the rest of the boundaries, perfectly matched layer (PML) conditions are used.

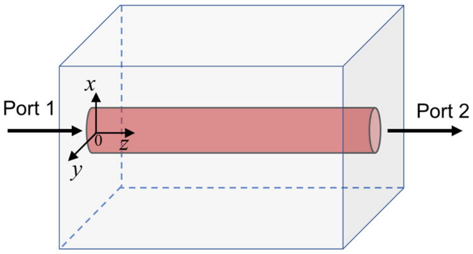

Fig. S11. Schematic plot of the configuration for the 3D simulation.

To verify the 3D model, Fig. S12 gives the calculated energy density distributions along the propagation direction of our proposed WS_2-clad MNW working at different wavelengths. Since the energy is highly concentrated in the 1-nm WS_2 cladding and the features in Fig. S12a(i-iii) are difficult to distinguish, we further normalize them and plot in a color bar with saturation [9] for better visualization (Fig. S12b(i-iii)). As are shown, when the wavelength approaches the excitonic resonance (616 nm), the loss of the PEPP dramatically increases, resulting in a sharply reduced propagation length (L_m), which coincides very well with our theoretical prediction in the manuscript. For further quantitative validation, we calculate L_m from the transmission S_{21} parameter between the output and input ports [8] based on our 3D simulation as:

$$L_m = L / (2\,\mathrm{In}|S_{21}|)\,. \tag{S5}$$

For λ=610, 616, 620 nm shown in Fig. S12, the calculated L_m are 909.7, 238.1, 399.0 nm respectively. Compared to the result from 2D calculation (L_m=935.9, 239.2, 400.8 nm for λ=610, 616, 620 nm, Fig. 4(b)), a good agreement between them can be achieved.

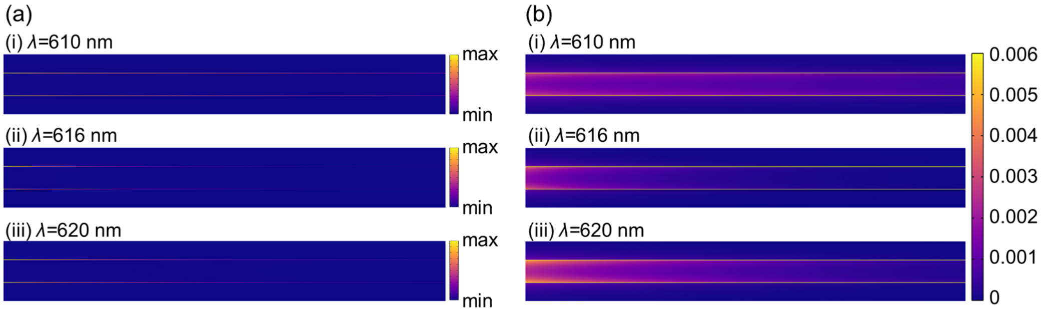

Fig. S12. (a) Energy density distributions along the propagation direction of a WS_2-clad MNW at the wavelength λ=610, 616, and 620 nm. (b) For better visualization, they are normalized and plotted in a color bar with saturation.

5. Extending the strong coupling strategy to other structures

Besides the WS_2-clad cylindrical MNW we demonstrated in the manuscript, the cavity-free strong coupling strategy can be further extended to other configurations such as a WS_2-clad pentagonal MNW (Fig. S13) and a bare MNW on a flat substrate with a WS_2 layer on top of it (Fig. S14), enabling large Rabi splitting (Fig. S13(a) and Fig. S14(a)) and tightly confined energy inside the WS_2 layer (Fig. S13(b) and Fig. S14(b)).

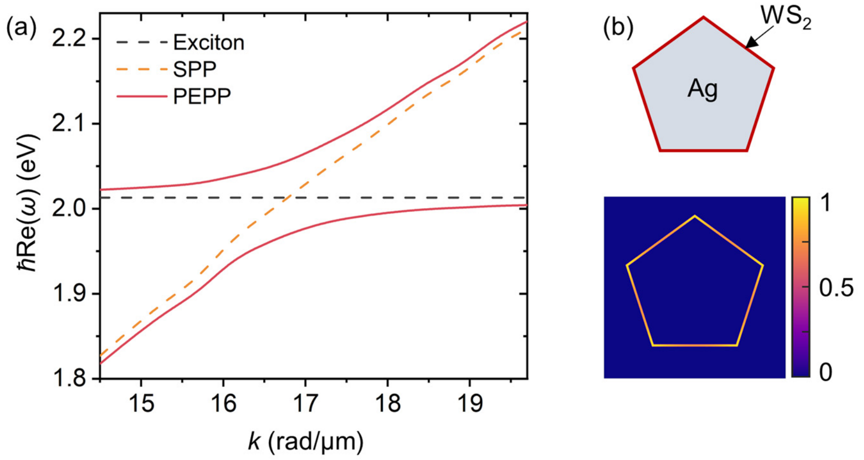

Fig. S13. Strong coupling in a 50-nm-diameter pentagonal MNW with a WS_2 cladding. (a) Rabi splitting dispersion and (b) mode profile.

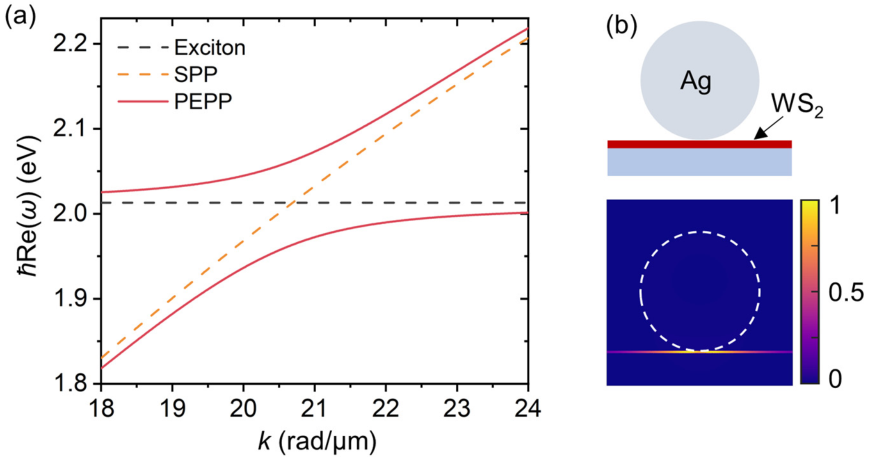

Fig. S14. Strong coupling in a bare MNW (50-nm diameter) placed on a flat silica substrate with a WS_2 layer on top of it (a) Rabi splitting dispersion and (b) mode profile.

References

1. L. Zhang, R. Gogna, W. Burg, E. Tutuc, and H. Deng, "Photonic-crystal exciton-polaritons in monolayer semiconductors," Nat. Commun. **9**(1), 713 (2018).
2. K. Kolwas and A. Derkachova, "Impact of the Interband Transitions in Gold and Silver on the Dynamics of Propagating and Localized Surface Plasmons," Nanomaterials **10**(7), 1411 (2020).
3. A. Canales, D. G. Baranov, T. J. Antosiewicz, and T. Shegai, "Abundance of cavity-free polaritonic states in resonant materials and nanostructures," J. Chem. Phys. **154**(2), 024701 (2021).
4. R. Ruppin, "Electromagnetic energy density in a dispersive and absorptive material," Phys. Lett.

A **299**(2–3), 309–312 (2002).
5. R. F. Oulton, V. J. Sorger, D. A. Genov, D. F. P. Pile, and X. Zhang, "A hybrid plasmonic waveguide for subwavelength confinement and long-range propagation," Nat Photon. **2**(8), 496–500 (2008).
6. S. A. Maier, *Plasmonics: Fundamentals and Applications* (Springer, New York, 2007).
7. J. Sun, Y. Li, H. Hu, W. Chen, D. Zheng, S. Zhang, and H. Xu, "Strong plasmon–exciton coupling in transition metal dichalcogenides and plasmonic nanostructures," Nanoscale **13**(8), 4408–4419 (2021).
8. Wave Optics Module User's Guide. COMSOL Multiphysics® v. 6.0. COMSOL AB, Stockholm, Sweden. 2021
9. Q. Li and M. Qiu, "Plasmonic wave propagation in silver nanowires: guiding modes or not?," Opt. Express **21**(7), 8587 (2013).
